# Rapid and Non-Enzymatic *In Vitro* Retrieval of Tumour Cells from Surgical Specimens

**DOI:** 10.1371/journal.pone.0055540

**Published:** 2013-01-31

**Authors:** Brigitte Mack, Carola Eggert, Katharina Eder, Sannia Imrich, Philipp Baumeister, Ulrich Harréus, Olivier Gires

**Affiliations:** Department of Otorhinolaryngology, Head and Neck Surgery, Grosshadern Clinic, Ludwig-Maximilians-University, Munich, Germany; University of Tübingen, Germany

## Abstract

The study of tumourigenesis commonly involves the use of established cell lines or single cell suspensions of primary tumours. Standard methods for the generation of short-term tumour cell cultures include the disintegration of tissue based on enzymatic and mechanical stress. Here, we describe a simple and rapid method for the preparation of single cells from primary carcinomas, which is independent of enzymatic treatment and feeder cells. Tumour biopsies are processed to 1 mm^3^ cubes termed explants, which are cultured 1–3 days on agarose-coated well plates in specified medium. Through incisions generated in the explants, single cells are retrieved and collected from the culture supernatant and can be used for further analysis including *in vitro* and *in vivo* studies. Collected cells retain tumour-forming capacity in xenotransplantation assays, mimic the phenotype of the primary tumour, and facilitate the generation of cell lines.

## Introduction

Nowadays, the great majority of knowledge related to tumour biology stems from or was verified in established tumour cell lines, which have at times been generated long ago and have been passaged innumerable times. Additionally, concepts in molecular oncology are addressed *in vivo* using sophisticated animal models of disease, *e.g.* transgenic and knockout mouse strains [1]. However, the use of animal models is time consuming and still requires verification of the unravelled phenotype in independent cell systems. Genetic and phenotypic changes, occurring in culture during selection for cells able to adapt to *in vitro* conditions, might impact on the expression and functionality of genes of interest and therefore potentially bias studies. Especially for the case of genomic analyses, divergences between primary cells and permanent lines might be of great importance and are controversially discussed [2]. This is even more pronounced in the case of tumours with phenotypically and molecularly heterogeneous sub-types, such as for example breast cancer. Here, tumour cell lines revealed genetically more complex than primary tumours, which renders the choice of representative cell lines imperative [3]. Hence, cell lines and primary cells have their advantages and disadvantages, which eventually complement each other. Cell lines represent a rather pure source of tumour cells, which might have acquired additional, cell culture specific modifications and/or lost primary cell-specific features. Primary tumour cells obviously reflect the *in vivo* situation in the human being more closely, but might contain contaminating non-tumour cells and are commonly more difficult to obtain in sufficient amounts. Hence, the comparison of primary cultures and long-term established cell lines is a matter of research, which however cannot be conducted in every given case. Nonetheless, the study of primary tumour cells is of utmost importance to obtain insights in molecular processes occurring *in situ* as closely as possible.

Commonly, the generation of short-term primary cultures is accompanied by enzymatic and/or mechanical treatment of cells [4]. Treatments with trypsin, collagenases, and DNAses follow the mechanical disruption of tissue integrity to release tumour cells from the extracellular matrix [5]. Alternatively, more complex methodologies such as centrifugation on Percoll gradients or micro-dissection of the cells of interest are in use [6]. We have concentrated on the isolation of primary tumour cells from surgical specimens of head and neck carcinomas without implementating a step of enzymatic digestion. The method relies on the incision of small explants from primary biopsies and the culture in supplemented Airway Epithelial Cell Growth medium, which is optimised for primary cells [7], [8]. Tumour cells were reproducibly retrieved in the culture medium within one to three days, and could be used for *in vitro* testing and re-implantation into immunocompromised mice. *In vivo*, single cells reconstituted tumours, which perfectly mimicked the human tumours of origin. In addition, cell lines, which express classical markers of carcinoma cell lines can be generated from explanted cells upon serial dilutions.

In summary, we present a rapid method to retrieve single tumour cells from primary carcinoma specimens without enzymatic treatment.

## Materials and Methods

### Generation of explants

Fresh primary specimens of head and neck carcinomas (n = 28) with a minimal size of 5 mm^3^ were processed within 30 min after surgical removal. Size refers to biopsies macroscopically devoid of fat tissue, musculature or any other surrounding, non-tumour tissue. All biopsies were obtained in accordance with the local ethics committee of the Ludwig-Maximilians-University (#426-11) and exclusively with written informed consent of each patient after education by surgeons in charge. Specimens were cut into 1 mm^3^ cube-like pieces using sterile Feather disposable scalpels N°11. These 1 mm^3^ cube-like pieces are referred to as explants. A total of three to five incisions were made with the point of the scalpel on the sides of the explants to generate small channels ranging crosswise through the explants. These incisions loosen the tissue structure and generate channels, which facilitate the exit of cells from explants. Explants and incisions were performed in a drop of NaCl solution (0.9% m/V) to avoid drying of explants. Maximally five explants were placed in one well of 24-well format plates or one explant per well in 96-well format plates. To do so, explants were harvested with 1 ml pipette tips and transferred into culture plate wells. Before usage the pipette tips were trimmed with a scalpel in order to enlarge the tip hole. Culture plates were coated beforehand with a 2% agarose (Biozym, ME Agarose, Hess. Oldendorf, Germany) solution in PBS with a 0.05% matrigel coat (Growth Factor Reduced BD Matrigel Matrix BD Biosciences, USA). 300 µl of agarose solution were used for a complete coverage of wells in 24-well format plates. Ice-cold matrigel was resuspended in ice-cold PBS to a final concentration of 0.05%, mixed vigorously, and was added as a thin layer (50 µl). Coated plates were stored at 4°C before use. In case of the generation of permanent cell lines, only 250 µl of agarose solution were used to keep a crescent-like area in the well, which was free of agarose and can serve as an adhesion platform for cells in the absence of matrigel. Explants were cultured in Airway Epithelial Cell Growth medium supplemented with SupplementMix C39165 (Promocell, Heidelberg, Germany) for one to seven days. The combination of small-sized explants, incisions, and specified cell culture medium containing chemoattractants warranted an efficient migration of cells out of explants into the supernatant. Cells were collected between days 1 to 3 based on microscopic control of the cell culture supernatants' content and subjected to further *in vitro* or *in vivo* analyses. Collection of cells was conducted upon resuspension and harvesting of the complete supernatant with 100 µl pipette tips in order to strictly avoid harvesting explants and to harvest all cells in suspension. Thereafter, cells were gently centrifuged at 0.3 relative centrifugal force (RCF) for 2 minutes and pellets were resuspended in PBS or cell culture medium, depending on further treatment.

### Generation of cell lines

To establish permanent cell lines, explants were plated on incompletely agarose-coated wells (see above). Cells, which had migrated out of explants, were allowed to adhere to uncoated areas for 5–7 days. Thereafter, adherent cells were detached from the culture well upon trypsin treatment with a 10 µl pipette tip and were transferred onto standard culture plates (Nunc, Langenselbold, Germany). Serial dilutions ranging from 1∶10–1∶1000 were prepared to eliminate fibroblasts contamination.

### Flow cytometry

Before flow cytometry measurement, cells were washed three times in FACS buffer (PBS containing 3% FCS). Cells were incubated with the EpCAM specific antibody Ber-Ep4 (Dako, Hamburg, Germany; 1∶50 in FACS buffer, 15 min). After centrifugation, supernatant was discarded and cells incubated with a fluorescein isothiocyanate (FITC)-conjugated secondary antibody (1∶50 in FACS buffer, 15 min). Cells were again spun and resuspended in PBS containing 3% FCS+1 µl PI (propidium iodide, 1 mg/ml). Cell surface expression of EpCAM was analysed in a FACS Calibur cytometer (Becton Dickinson, Heidelberg, Germany). Control staining was performed using a mouse IgG1 isotype control antibody (Dako, Hamburg, Germany). Immunocharacterisation of one established cell line referred to as PiCa was performed with the following antibodies: CD11a, CD11c, CD14, CD16, CD28, CD40-L, CD45-RA, CD56, EGF-R, EpCAM/Ber-Ep4, ICAM-1, IL2-R, integrin α4β1, VCAM-1 (all Dako, Hamburg Germany), CCR5 (R&D Systems, Minneapolis, USA), CD133 (Miltenyi, Bergisch Gladbach, Germany), CD20, N-cadherin (both Sigma Aldrich, Taufkirchen, Germany), CD44 (Dianova-Immunotech, Hamburg, Germany), CD90 (DCS, Ha mburg, Germany), CD95/Fas, Fas-L (both BD Pharmingen, Heidelberg, Germany), E-cadherin (Cell Signaling), integrin β3, SSEA-1, VEGF (all Santa Cruz, Heidelberg, Germany), MHC class 1 (Epitomics, Burlingame, USA), MHC class 2 (Oncogene Science, Wilex AG, Munich Germany), Mucin 1 (Novo Castra, Wetzlar, Germany).

### Immunocytochemistry and immunohistochemistry

Immunocytochemistry and immunohistochemistry were performed as described [9] using the EpCAM-specific antibody Ber-Ep4 and the Ki-67-specific clone Ki-67 antibody (both Dako, Hamburg, Germany).

### Transient transfection, plasmids, siRNAs

PiCa cells were transiently transfected using MATra (Iba, Goettingen, Germany) or Fugene (Promega, Mannheim, Germany) reagents according to the manufacturers' protocol. The peGFP-C1 expression plasmid from Clontech (Mountain View, USA) was used for transient transfections. The following siRNA oligonucleotides were used for the transient knock-down of EpCAM in PiCa cells:

- EpCAM-specific siRNA: (5′-UGCCAGUGUACUUCAGUUG-3′),
- control siRNA: (5′-UCGUCCGUAUCAUUUCAAU-3′).

### Xenotransplantation in immunocompromised mice

All experiments were performed with the approval of the Ethics Commission of the Ludwig's Maximilian University Munich (Az.55.2-1-54-2532-101-07). Primary cells or PiCa cells (1×10^4^–5×10^6^ cells per injection in 100 µl medium and 100 µl Growth Factor Reduced BD Matrigel Matrix BD Biosciences, California, USA) were injected in the flanks of 6–8 week old NOD/SCID mice. After tumour development (latest 28 days after injection), mice were sacrificed by isofluran inhalation, tumour weight was assessed using a precision weighting scale and tumour tissues were frozen for immunohistochemical analyses.

## Results

### Short-term explantation of tumour cells from primary head and neck carcinoma specimens

**Figure 1:**
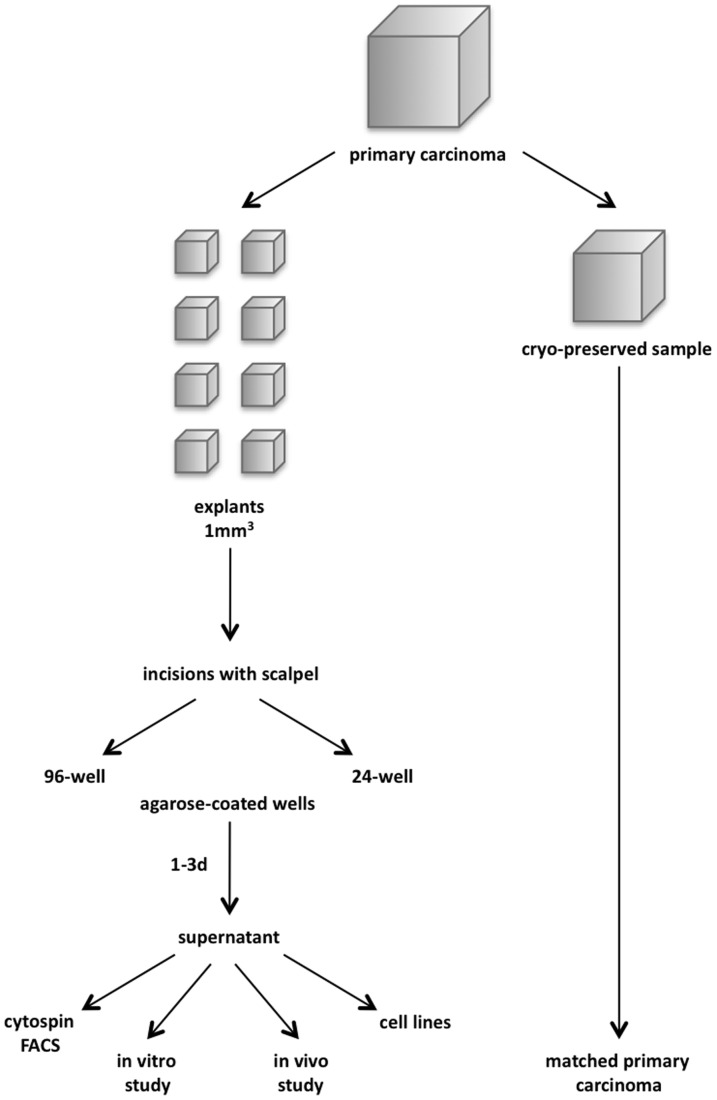
Schematic representation of the method of tumour explant formation. Specimens of primary carcinomas (>5 mm^3^) are cut into approximately 1 mm^3^ cube-like structures termed explants. One part of the primary carcinoma is cryo-preserved as a matched control for explanted cells. Incisions are generated in explants, which are then transferred into agarose-coated 96- or 24-well plates for a time period of 1–3 days. Supernatants are collected and cells harvested upon centrifugation for subsequent analysis or examination.

**Figure 2:**
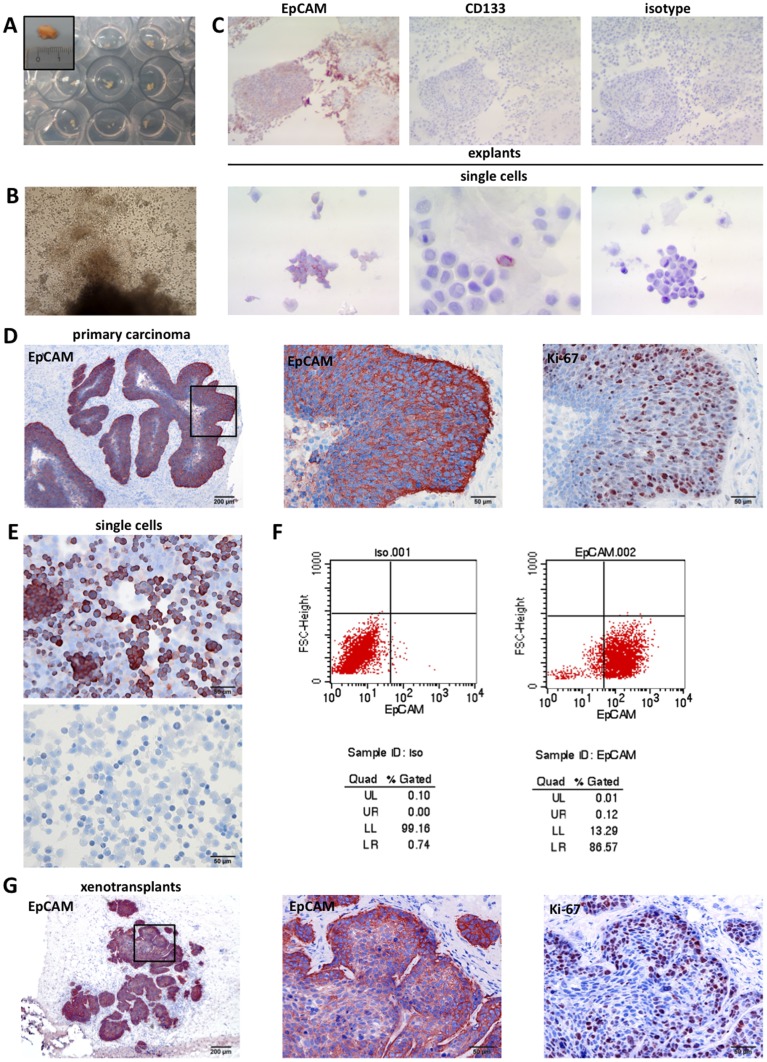
Single cells from head and neck specimens recapitulate primary tumours. (**A**) Biopsy of a hypopharynx carcinoma was processed to explants of approx. 1 mm^3^ and transferred into agarose-coated 24-well plates. Shown is the primary tumour biopsy and explants in wells. (**B**) Upon incision and cultivation in specified medium, cells migrate out of the explant and settle in the well. Shown is one representative example at day one. (**C**) Upper panels: Explants were collected after one day, cryo-preserved and processed to sequential sections before staining with EpCAM-, CD133-specific or an isotype control antibody as indicated. The structure of explants begins to disintegrate and cells migrate away from the major tumour area and are released into the supernatant. Cells express EpCAM but no CD133. Lower panels: Single cells migrating out of explants were subjected to an EpCAM-, CD133 or isotype control antibody staining. The majority of cells expressed EpCAM and only rarely expressed CD133. (**D**) Matched primary carcinoma was stained for the expression of EpCAM and Ki-67. Shown are 40× (upper panel) and 200× magnifications (middle and lower panels) of the indicated stainings. (**E**) Single cells from explants of primary specimen shown in (D) were stained in cytospins with EpCAM-specific (upper panel) or an isotype control antibody (lower panel). (**F**) Cell surface expression of EpCAM on single cells from explants of primary specimen shown in (D) was analysed upon flow cytometry with specific antibodies. Left dot plot represents isotype control, while right dot plot represents EpCAM staining. Dot plot statistics according to the marker set for both graphs are given for isotype control and EpCAM staining. (**G**) Single cells (1–5×10^6^) from explants of primary specimen shown in (D) were xenotransplanted subcutaneously in immunocompromised NOD-SCID mice. After 28 days tumours were surgically removed, cryo-preserved, and stained for the expression of EpCAM and Ki-67. Shown are 100× (upper panel) and 200× magnifications (middle and lower panels) of the indicated stainings.

Primary tumour specimens from various localisations of the head and neck area (Table 1) were subjected to a rapid explantation method with the aim to retrieve single tumour cells. Biopsies were first cut into approximately 1 mm^3^-sized cubes (termed explants) into which three to five incisions were made. These explants were then transferred onto agarose-coated 96- or 24-well plates and cultured in supplemented Airway Epithelial Cell Growth medium. Supernatants of each well were collected after one to three days. To compare explants with matched primary tumours, a sample of each tumour was cryo-preserved at the beginning of the procedure. Cells present in supernatants were used for cytospin and flow cytometry-assisted stainings, *in vitro* and *in vivo* studies, and eventually to establish permanent cell lines (schematic representation in Figure 1). Tumour biopsies had to be at least 5 mm^3^ in size to retrieve sufficient tumour cells for further analysis. This size refers to biopsies macroscopically devoid of fat tissue, musculature or any other surrounding, non-tumour tissue. Figure 2A exemplifies the generation of explants from a tongue carcinoma biopsy into 24-well format culture plates. Within one day, cells migrated out of the explant and remained at first in the culture supernatant without adhesion owing to agarose coating (Figure 2B and Figure S1A). Staining of cryo-preserved explant samples show a staining for the pan-epithelial marker EpCAM and rare or no staining for the pentaspan cell surface protein CD133 (Figure 2C, upper panels). In these explants, enzyme-independent dissociation and loss of structure were seen over the time course of three days (Figure S1B). Cells that had migrated out of the explant into the supernatant were collected and subjected to immunostaining of cytospins on glass slides. Single cells expressed EpCAM in the majority of cells, while the cancer stem cell marker CD133 was restricted to rare single cells (Figure 2C, lower panels). Further immunocytochemical analyses revealed that >80% of cells in the supernatants of explants were positive for the epithelial marker pan-cytokeratin, whilst negative for the fibroblast marker 5B5 (Figure S1C).

**Table 1:** Primary carcinomas used for the generation of explants.

Tumour	IHC EpCAM	EpCAM MFI-R d1	EpCAM MFI-R d2–5	Mean MFI-R
tonsillar	+++	8.3	4.7	6.5
soft palate	+++	n.d.	4.8	4.8
floor of the mouth	+++	n.d.	7.5	7.5
oropharynx	+++	6.3	16.2	11.25
oropharynx	+++	4.4	2	3.2
CUP	+++	4.2	4.2	4.2
larynx	+++	6.5	5.7	12.2
tonsillar	+++	4	2.9	3.45
tonsillar	+++	4.2	0.3	2.25
hypopharynx	+++	4.9	n.d.	4.9
hypopharynx	+++	22.4	n.d.	22.4
tonsillar	+++	0.8	n.d.	0.8
tonsillar	++	17.5	2.3	9.9
tonsillar	++	2.3	2.1	2.2
oropharynx	++	1.5	5.5	3.5
oropharynx	++	n.d.	1.7	1.7
oropharynx	++	0.8	1.3	1.05
soft palate	++	1.8	1	1.4
larynx	++	n.d.	0.7	0.7
hypopharynx	++	n.d.	1.4	1.4
base of tongue	++	2.7	3	2.85
oropharynx	++	2.1	0.8	1.45
larynx	−/+	3.4	n.d.	3.4
larynx	−	n.d.	1.3	1.3
oropharynx	+	0.9	1.6	1.25
tongue	+	1.2	1.1	1.15
oropharynx	+	1.5	1.5	1.5
larynx	+	1.1	1.6	1.35

The indicated primary carcinomas were processed to explants and collected cells were analysed for the expression of EpCAM upon immunohistochemistry (IHC) and flow cytometry with EpCAM-specific antibodies. MFI-R: mean fluorescence intensity ratio (EpCAM/isotype control antibody). Staining intensities represent +++: strong; ++: intermediate; +: weak; −: negative. n.d.: not determined.

Out of 28 specimens, EpCAM staining upon immunohistochemistry (IHC) of primary tumour biopsies varied: 12 carcinomas showed positive staining for EpCAM in immunohistochemistry (IHC) stainings of the primary tumours, while 10 carcinomas expressed intermediate levels and 6 carcinomas expressed low levels or no EpCAM (Table 1). Single cells from explants of these specimens were assessed for the expression of EpCAM upon FACS analysis at day 1 and between day 2 to 5. Except for one tonsillar, one oropharynx, and one soft palate carcinoma, which did not allow explantation of EpCAM-positive single cells, EpCAM-positive single cells could be retrieved from all primary specimens expressing EpCAM to strong or intermediate levels (Table 1). Specimens showing a low or negative expression level of EpCAM in IHC, yielded single cells matched expression levels (Table 1). Numbers of vital cells retrieved from explants were assessed systematically at day 1–2 in six specimens and ranged from 6.4×10^2^ to 5×10^5^ per 1 mm^3^. The vitality of cells ranged from 17% to 81% and the expression of CD133 between 0% and 0.6%. In case of longer cultivation of explants, the numbers of cells retrieved in the supernatant decreased, however the vitality of cells increased up to 100%.

### Single cells from short-term explants are tumourigenic *in vivo*

In an attempt to further characterise the tumourigenic potential of single cells from explants, we have processed a biopsy of a hypopharynx carcinoma to explantation. Primary carcinoma cells strongly expressed EpCAM especially at the tumour leading edges, which correlated with areas of proliferation as monitored upon Ki-67 staining (Figure 2D). This correlation of EpCAM and Ki67 staining confirmed earlier findings related to the regulation of proliferation and expression of Cyclin D1 by EpCAM [10]. Although being of squamous nature, this hypopharynx carcinoma grew in an adenocystic manner forming lobules and gland-like structures (Figure 2D). Cytospins of single cells showed a specific expression of EpCAM in approximately 70–80% (Figure 2E), which was confirmed upon FACS staining (86%, Figure 2F). A single cell suspension of 1–5×10^6^ cells was injected subcutaneously into the flank of immunocompromised NOD-SCID mice (n = 2) in a 1∶1 dilution in matrigel. Tumour formation was assessed upon palpation on a daily basis. Within 28 days, xenotransplanted single cells generated tumours with a morphology and EpCAM expression pattern similar to the human carcinoma of origin. Xenotransplants grew in a focal, gland-like manner and expressed EpCAM and Ki-67 in a correlated manner at the edges of tumour areas (Figure 2G). Comparably to the primary specimen, EpCAM expression was strongest in areas of strong proliferative activity as monitored upon the expression of Ki-67 (Figure 2G, middle and lower panels).

### Single cells from short-term explants allow for the establishment of permanent cell cultures *in vitro*

**Figure 3:**
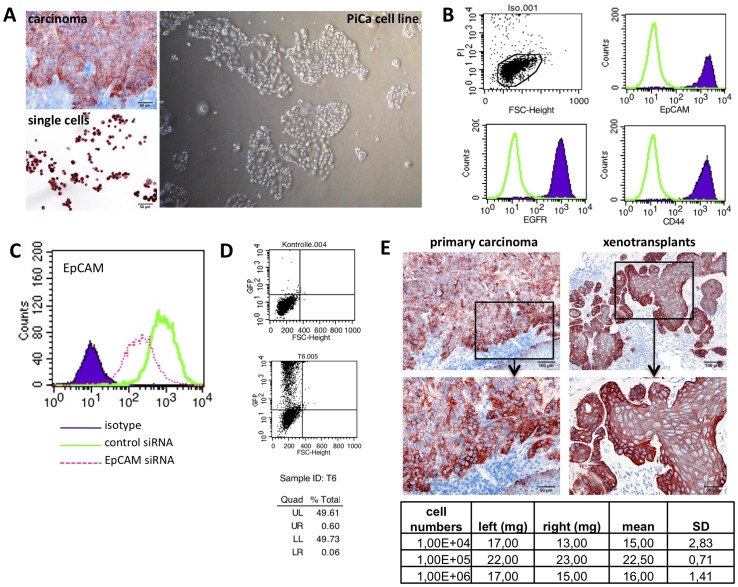
Generation of a permanent cell line from tumour explants. (**A**) Matched sample (upper left panel) and single cells (lower left panel) obtained from explants of a larynx carcinoma were stained for the expression of EpCAM. Harvested cells were passaged to establish a permanently growing cell line. PiCa cells grew in adherent manner and displayed heterogeneity in morphology (right panel). (**B**) PiCa cells were monitored for the cell surface expression of EpCAM, EGF-R, and CD44 using specific antibodies. Cells represented one determined population (FSC) and were vital (PI) as shown in the dot plot graph (upper left). (**C**) PiCa cells were transiently transfected with control or EpCAM-specific siRNA. After 24 hrs, cells were analysed for the cell surface expression of EpCAM upon flow cytometry. Isotype control staining is depicted as solid, purple curve, control siRNA as green line, and EpCAM-siRNA as dashed pink line. (**D**) PiCa cells were transiently transfected with the eGFP-C1 expression plasmid. After 24 hrs, expression of GFP was assessed upon flow cytometry. Dot plots graphs represent control transfectants (upper panel) and GFP transfectants (lower panel). Quadrants were set according to control transfectants and statistics are given below. (**E**) PiCa cells (1×10^4^, 1×10^5^, 1×10^6^) were subcutaneously injected into the left and right flanks of immunocompromised NOD-SCID mice. After 21 days, tumours were surgically removed, cryo-preserved and stained for the expression of EpCAM. Matched primary specimens were stained in parallel (left panels) to xenotransplants (right panels) and are depicted in 100× and 200× magnification. Injected cell numbers and tumour weights with mean and standard deviations are given in tabular manner.

Next, single cells were retrieved from larynx carcinoma biopsy, which displayed an intermediate to strong expression of EpCAM in approximately 95–100% of cells (Figure 3A, upper left panel). This expression pattern of EpCAM was similarly observed in explanted single cells in culture supernatant (Figure 3A, lower left panel). Fibroblasts migrating out of explants were observed after 7–14 days, hence single cells , which had adhered to the spared agarose-free area, were harvested at day 7. Serial dilutions in 24-well format were performed in order deplete remaining fibroblasts. The resulting cell line termed PiCa permanently grew in culture in supplemented Airway Epithelial Cell Growth medium. In order to adapt PiCa cells, serial diluation of Airway Epithelial Cell Growth medium with HAM's F12/M199 medium and 10% fetal calf serum was used on a weekly basis to achieve a full replacement of Airway Epithelial Cell Growth medium. PiCa cells cultured in monolayers displayed primarily cells characterised by an epithelial, cobblestone-like phenotype, interspersed with bigger and more flattened cells regularly observed in permanent cultures of head and neck carcinomas such as FaDu and PCI cells (Figure 3A, right panel). The expression of various cell surface markers associated with different cell lineages was assessed on PiCa cells upon FACS analysis with specific antibodies. PiCa cells strongly expressed markers of epithelial cells including EpCAM, EGF-R, CD44 (Figure 3B), ICAM-1, and Mucin-1 (Table 2), along with more ubiquitous markers such as CD95 (Fas) and MHC class 1 (Table 2). PiCa cells did not express markers of immune cells (CD20, CD28, CD40-L, CD45RA, CD56, CD90, IL2-R), nor of macrophages/monocytes (CCR5, CD11a, CD11c, CD14, CD16) (see Table 2 for detailed immune reactivity).

**Table 2:** The indicated cell surface markers were measured in three independent experiments with specific antibodies using flow cytometry.

Marker	MFI ratio	SD
CCR5	1.1	0.3
CD11a	1.1	0.2
CD11c	1.0	0.2
CD133	1.0	0.2
CD14	1.8	1.1
CD16	1.0	0.2
CD20	1.1	0.4
CD28	0.8	0.1
CD40-L	0.9	0.1
CD44	58.1	45.2
CD45RA	1.0	0.2
CD56	1.0	0.2
CD90	0.9	0.2
CD95 (Fas)	5.2	1.9
E-Cadherin	1.3	0.0
EGF-R	33.5	7.5
EpCAM	84.5	34.5
Fas-L	1.3	0.3
ICAM-1	11.7	7.4
IL2-R	1.0	0.4
Integrin α4β1	1.1	0.2
Integrin β3	1.9	1.3
MHC class 1	2.9	0.7
MHC class 2	1.5	0.7
Mucin-1	11.3	4.4
N-Cadherin	1.1	0.2
SSEA-1	6.4	5.5
VCAM-1	1.1	0.2
VEGF	1.2	0.2

Shown are mean fluorescence intensity ratios (MFI ratio) and standard deviations (SD).

Next, we tested the feasibility of PiCa cells to be extrinsically modulated with respect to the expression of endogenous proteins. PiCa cells were transiently transfected with small inhibitory RNA molecules (siRNAs) specific for the tumour-associated antigen EpCAM or with control siRNAs. After 24 h, the expression levels of EpCAM were addressed upon FACS analysis. Transient transfection of EpCAM-specific siRNA resulted in a maximal knock-down efficiency of 72% of EpCAM protein expression at the cell surface (Figure 3C). The efficiency of a transient transfer of expression plasmids into PiCa cells was monitored with a plasmid encoding the green fluorescence protein GFP and using two different transfection methods, *i.e.* MATra and lipofection. After 24 h, nearly 50% of PiCa cells expressing GFP were detected upon FACS measurement (Figure 3D).

The tumourigenicity of PiCa cells was assessed *in vivo* in immunocompromised NOD-SCID mice in a pilot experiment. Three concentrations of PiCa cells (1×10^4^, 1×10^5^, 1×10^6^ cells per injection) were subcutaneously injected into both flanks of NOD-SCID mice (n = 1 for each cell concentration). Tumour formation was assessed upon daily palpation and tumour weight measured after surgical excision of tumours at day 21. Similarly to the primary specimen of origin, PiCa cells established carcinomas characterised by a focal growth and a strong expression of EpCAM at the edges of tumour areas (Figure 3E). Each of the chosen PiCa cell concentrations generated tumours *in vivo* with weights ranging from 13 to 23 mg (Figure 3E, lower table). Thus, PiCa cells retained a tumour phenotype similar to the specimen of origin.

## Discussion

Functional analyses of molecules involved in processes of cancer comprises three types of experimental systems: animal models of cancer [11], primary short-term cultures of tumour cells *ex situ*, and permanent cancer cell lines [12]. Each system displays advantages and disadvantages, which are inherent to the methods used. Knowledge about the actual situation in patients is a prerequisite to fully understand underlying mechanisms of tumourigenesis and, eventually, to set up efficient therapeutic modalities such as chemotherapy and adjuvant immunotherapy. Primary cancer cells from patients analysed *ex situ* represent closest the *in vivo* situation, but require a rapid method for the isolation of cancer cells owing to the short-term culture opportunities and the associated necessity for repeated experiments. Conventional methods to isolate primary tumour cells from human biopsies rely on enzymatic disaggregation and mechanical mincing [4], [13]. Although functional, enzymatic treatment may not only degrade the extracellular matrix (ECM) and DNA, but might also affect tumour cells themselves, e.g. via the partial degradation of trypsin-sensitive substrates. Isolation of lymphocytic cells from the liver with or without treatment with collagenase yielded different cell types [14], pointing towards an influence of enzymatic treatment on the capacity of cells to emigrate from tissues. Accordingly, it was advised to adapt the time of exposure to ECM-degrading enzymes for each tumour type in order to achieve a partial enzymatic degradation [13].

Therefore, we have developed an alternative method to isolate tumour cells from primary cancer biopsies without a need for enzymatic treatment. To facilitate the dissociation and emigration of tumour cells from the explant into the cell culture medium, we chose small sizes of explants (1 mm^3^), generated incisions in these explants, which provide channels for cells to exit explants, and cultivated explants in supplemented Airway Epithelial Cell Growth medium, which is adapted for primary cells [7], [8], [15]. Cryosections of explants at various time points indicated a progressive disintegration of these structures and a relief of EpCAM-positive cells from the bulk. Although the precise molecular mechanism of disintegration of extracellular matrix by cells emigrating from explants is unknown, the contribution of membrane-associated proteases such as matrix metalloproteinases is to be expected [16]. The great majority of cells emigrating from explants expressed the signalling active membrane protein EpCAM, which is an activator of MMP7 and might thereby contribute to single cell emigration [17]. This is corroborated by stainings of cryo-preserved explants at succeeding time points, which demonstrated an ongoing loosening of tissue structures mostly around EpCAM-expressing cells. Furthermore, microscopic observation of explants *in vitro* disclosed a preferential emigration of cells at distinct sites, supposedly representing the incisions previously generated (Figure S1).

Cells, which migrated out of the explant were mostly vital, sufficient in numbers for subsequent *in vitro* and *in vivo* studies, and expressed markers of epithelial origin (EpCAM, cytokeratins) but not fibroblast (5B5). The tumourigenic capacity of single cells that had migrated out of explants was confirmed *in vivo* upon xenotransplantation into immunocompromised mice. Importantly, short-term isolates of tumour cells recapitulated the phenotype of matched cryo-preserved samples, thus suggesting that they closely resemble the primary tumours of origin and pheno-copy them upon xenotransplantation. The short time between biopsy procedure, single cell retrieval, and subsequent analysis *in vitro* and *in vivo* does not allow for ample genetic alterations. This might particularly add to the stability of phenotype and the reliability of the method. Moreover, a hallmark of the presented method is the lack of enzymatic treatment of cells, which contributes to preserve the surface integrity and antigen expression pattern of tumour cells. Obviously, this method will to a certain degree lead to a preferential isolation of cells with migratory and tissue remodelling capacities. However, microscopic views of explants at late stages of in vitro culture disclosed a virtually complete lack of cells within the remaining structures.

Slight methodological changes to the protocol allowed for the establishment of a permanent cell line, which was subsequently adapted to standard culture media and could be used *in vitro* and *in vivo* in mouse models. Again, these cells pheno-copy the primary tumour of origin closely and generate tumours in immunocompromised mice, which are indistinguishable from primary cancers, and thus provide valuable sources for tumour cell studies.

In summary, we present a rapid and facile method for the retrieval of vital tumour cells from primary carcinoma biopsies, which are of great use for the further study *in vitro* or *in vivo* in animal models. With this method, multiple testing and/or reproduction of experiments with primary tumours is feasible in short time, without a need for additional, costly devices, and without the influence of enzymatic treatment.

## Supporting Information

Figure S1 ***In vitro*** **characterisation of explants.** (**A**) Single cells migrate out *in vitro* through incision in explants. (**B**) Overview of the structure of explants *in vitro* at day 0, 2, and 3. Explants were stained for the expression of EpCAM at the indicated time points. The structure of explants disintegrates over time, releasing EpCAM-positive cells from the united cell structure. (**C**) Single cells, which have migrated out of explants *in vitro*, strongly express cytokeratins and lack the fibroblast marker 5B5. Shown are representative examples.
(TIFF)
