# Supplementary figures and images for: Rapid and Non-Enzymatic In Vitro Retrieval of Tumour Cells from Surgical Specimens
Source: PLoS One. 2013 Jan 31;8(1):e55540. doi: 10.1371/journal.pone.0055540 (PMC3561176; doi:10.1371/journal.pone.0055540)

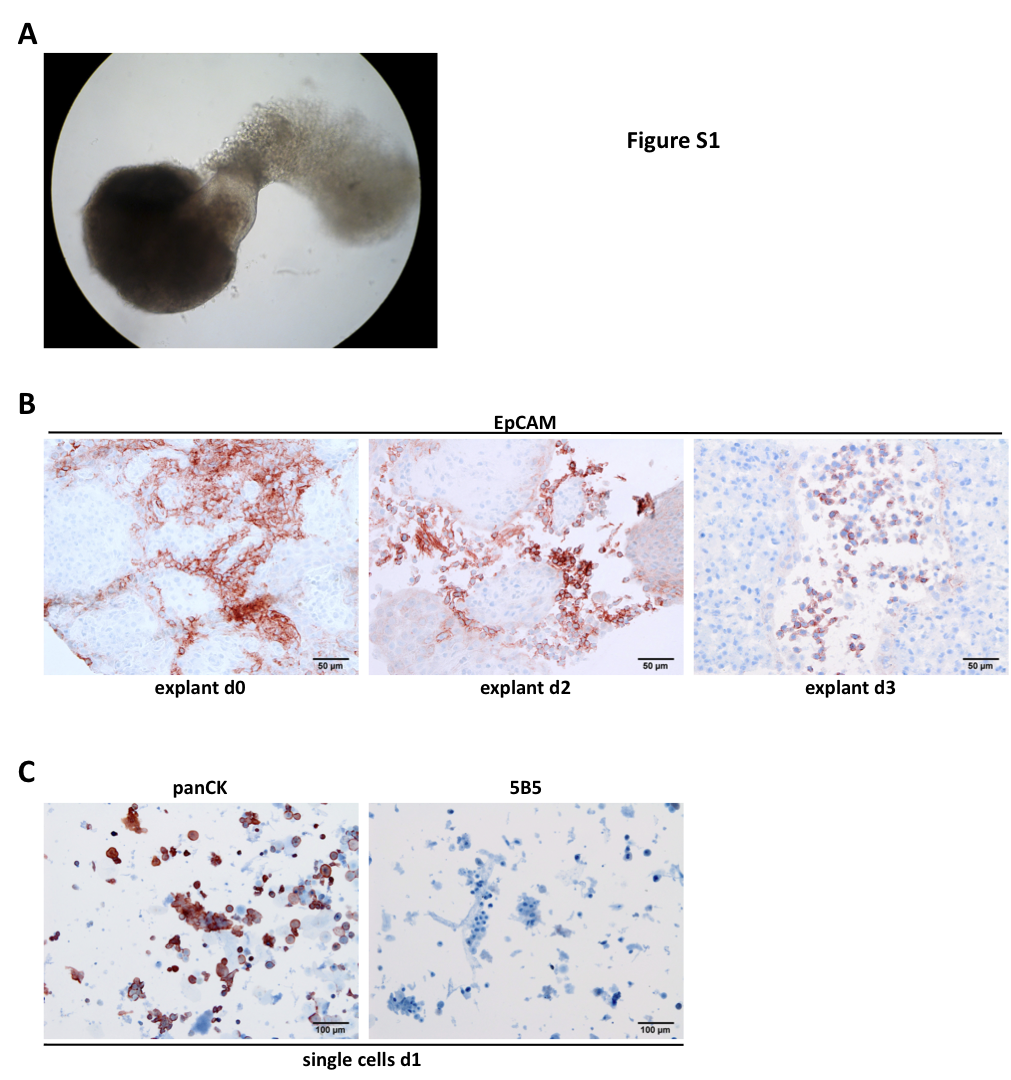

Supplement: Figure S1 — In vitro characterisation of explants. (A) Single cells migrate out in vitro through incision in explants. (B) Overview of the structure of explants in vitro at day 0, 2, and 3. Explants were stained for the expression of EpCAM at the indicated time points. The structure of explants disintegrates over time, releasing EpCAM-positive cells from the united cell structure. (C) Single cells, which have migrated out of explants in vitro, strongly express cytokeratins and lack the fibroblast marker 5B5. Shown are representative examples. (TIFF) [file pone.0055540.s001.tiff]
